# A Noisy Signal? Geographic Bias in FAERS Reports Linking Paracetamol to Autism Spectrum Disorder

**DOI:** 10.3390/jcm15020902

**Published:** 2026-01-22

**Authors:** Hülya Tezel Yalçın, Nadir Yalçın, Karel Allegaert, Pınar Erkekoğlu

**Affiliations:** 1 Department of Pharmaceutical Toxicology, Faculty of Pharmacy, Hacettepe University, 06230 Ankara, Türkiye; hulya.tezel@hacettepe.edu.tr (H.T.Y.); erkekp@hacettepe.edu.tr (P.E.); 2 Department of Clinical Pharmacy, Faculty of Pharmacy, Hacettepe University, 06230 Ankara, Türkiye; 3 Clinical Pharmacology and Pharmacotherapy, Department of Pharmaceutical and Pharmacological Sciences, Katholieke Universiteit Leuven, 3000 Leuven, Belgium; karel.allegaert@kuleuven.be; 4 Department of Development and Regeneration, Katholieke Universiteit Leuven, 3000 Leuven, Belgium; 5 Department of Hospital Pharmacy, Erasmus University Medical Center, 3015 GD Rotterdam, The Netherlands

**Keywords:** autism spectrum disorder, paracetamol, acetaminophen, prenatal exposure, developmental neurotoxicity, pharmacovigilance

## Abstract

**Background/Objectives:** Recent public and scientific discussions have raised concerns about a possible link between prenatal paracetamol exposure and autism spectrum disorder (ASD). However, pharmacovigilance-based evidence remains scarce, and the role of reporting bias has not been systematically assessed. This study aimed to characterize ASD-related adverse event reports involving paracetamol in the U.S. Food and Drug Administration’s Adverse Event Reporting System (FAERS) and to evaluate potential disproportionality signals, considering demographic, temporal, and geographic patterns. **Methods:** FAERS data from January 2010 to September 2025 were screened for reports listing paracetamol as the suspect drug and ASD-related Preferred Terms. After excluding duplicates and concomitant drugs, 1776 unique cases were analyzed. Patient demographics, reporter type, and country of origin were summarized descriptively. Disproportionality was calculated using four algorithms: Reporting Odds Ratio (ROR), Proportional Reporting Ratio (PRR), Information Component (IC), and Empirical Bayes Geometric Mean (EBGM). **Results:** Among 172,129 paracetamol-associated reports, 1776 (1.03%) included ASD-related terms. All were classified as serious and mostly submitted by consumers (98.6%). Gender was available in 47.7% of cases, showing male predominance (68.8%). Most reports referred to fetal exposure during pregnancy. Nearly all originated from the United States (98.4%). A marked rise was observed after 2022, with 562 reports in 2023 and 1051 in 2025. Disproportionality analyses revealed consistently elevated signals (ROR = 69.8, PRR = 69.2, IC025 = 5.60, EB05 = 48.3). **Conclusions:** The strong disproportionality signal likely reflects increased public attention and reporting dynamics rather than a causal association. Further integration of pharmacovigilance and epidemiologic data is warranted to clarify the clinical significance of these findings.

## 1. Introduction

Autism spectrum disorder (ASD) comprises a range of early-onset neurodevelopmental conditions characterized by persistent deficits in social communication, restricted and repetitive behaviors, and sensory anomalies. The Diagnostic and Statistical Manual of Mental Disorders, Fifth Edition, Text Revision (DSM-5-TR, 2022) consolidated prior subtypes—autistic disorder, Asperger’s syndrome, and pervasive developmental disorder not otherwise specified (PDD-NOS)—under a single spectrum diagnosis without major changes in diagnostic criteria [1].

ASD represents a major and growing global public health concern. Recent reports by the World Health Organization (WHO) and the U.S. Centers for Disease Control and Prevention (CDC) indicate a steady rise in prevalence worldwide, with notable regional variation across continents [2,3]. These trends reflect improved diagnostic capacity and persistent disparities in early detection and intervention [4].

The etiology of ASD is multifactorial, involving complex genetic, epigenetic, and environmental interactions. Several key genes implicated in ASD include SH3 and multiple ankyrin repeat domains (SHANK), neurexin (NRXN), contactin-associated protein-like 2 (CNTNAP2), and neuroligin (NLGN), which are involved in synaptic scaffolding, chromatin remodeling and transcriptional regulation [5]. Epigenetic studies reveal altered DNA methylation, histone modification, and non-coding RNA activity in ASD [6]. Environmental contributors—including maternal infection, fever, air pollution, and exposure to heavy metals, pesticides, phthalates, bisphenol A, or other pollutants [7,8,9,10]—along with maternal metabolic disorders (e.g., obesity, diabetes, metabolic syndrome) [11], may influence neurodevelopment via oxidative stress, mitochondrial dysfunction, endocrine disruption, and neuroinflammatory signaling [12,13,14,15]. Recent evidence suggests that both the dose and duration of prenatal exposure to certain environmental agents may modulate the risk of neurodevelopmental disorders, emphasizing the importance of quantifying exposure in epidemiologic analyses [16].

Diagnosis remains clinical and behavioral, relying on standardized tools such as the Autism Diagnostic Observation Schedule (ADOS) and Autism Diagnostic Interview–Revised (ADI-R). No pharmacologic therapy exists for the core symptoms; treatment focuses on behavioral and educational interventions, with medications such as risperidone and aripiprazole approved only for irritability [3].

### 1.1. Paracetamol in Pregnancy: Clinical and Regulatory Context

Paracetamol (acetaminophen) is the most commonly used analgesic and antipyretic in pregnancy. Historically classified by the U.S. Food and Drug Administration (FDA) as Pregnancy Category B—indicating no fetal risk in animal studies but a lack of adequate human data—it is now described under the Pregnancy and Lactation Labeling Rule (PLLR). The PLLR replaced letter categories with narrative summaries integrating human and animal data, pharmacologic mechanisms, and counseling recommendations [17]. The American College of Obstetricians and Gynecologists (ACOG) continues to endorse paracetamol as first-line therapy when clinically indicated, emphasizing the lowest effective dose and shortest duration as a general rule, while acknowledging that epidemiologic associations with neurodevelopmental outcomes do not prove causality [18]. Consistent with these, a recent meta-analysis reported that paracetamol use during pregnancy was not associated with an increased risk of autism spectrum disorder when based on physician-confirmed diagnoses (OR = 1.10, 95% CI = 0.98–1.24; E-value = 1.43) [19].

Although paracetamol is generally regarded as safe, concern persists about possible neurodevelopmental effects of prenatal exposure. Experimental and biomonitoring studies show efficient placental transfer and measurable fetal exposure. Ex vivo perfusion studies demonstrate approximately 45% maternal-to-fetal passage within an isolated placental perfusion unit [20], while physiologically based pharmacokinetic (PBPK) models predict fetal area under curve (AUC) values exceeding 90% of maternal levels [21]. Paracetamol and its conjugates (paracetamol-glucuronide, paracetamol-sulfate, paracetamol-cysteine and N-acetylcysteine conjugate) are detectable in >95% of cord-blood samples [22]. Animal experiments demonstrate that fetal brain/plasma ratios reach approximately 0.6 after single maternal dosing and exceed 1.0 after repeated exposure, while neonatal cerebrospinal fluid (CSF) levels remain comparable to plasma, indicating substantial permeability of the immature blood–brain barrier [22,23].

### 1.2. Mechanistic Basis for Developmental Neurotoxicity

Preclinical and mechanistic frameworks propose that prenatal paracetamol exposure may influence neurodevelopment through several interrelated biological pathways. These include fetal exposure via placental transfer, formation of reactive metabolites such as N-acetyl-p-benzoquinone imine (NAPQI) with subsequent glutathione depletion and oxidative stress, mitochondrial dysfunction with impaired redox homeostasis, and downstream modulation of neuroinflammatory processes and neurotransmitter systems. Although these mechanisms offer biological plausibility, direct human evidence—particularly from fetal brain tissue or well-characterized longitudinal neurodevelopmental follow-up studies—remains limited [24,25,26].

Preclinical findings further suggest that both the dose and duration of prenatal paracetamol exposure may modulate neurodevelopmental outcomes [16,27]. However, a recent quantitative weight-of-evidence (QWoE) review of preclinical studies questioned the translational relevance of these mechanistic hypotheses to human pregnancy, concluding that there is no consistent evidence of adverse neurodevelopmental effects at therapeutically relevant exposure levels [28]. In line with this assessment, no clear dose–response relationship has been established, and the proposed toxicity pathways may predominantly reflect suprapharmacological experimental conditions rather than clinically meaningful exposure scenarios [28,29].

### 1.3. Pharmacovigilance Rationale

Large-scale pharmacovigilance-databases (such as FAERS, Vigibase, EudraVigilance) [30] may provide a complementary perspective given the pervasive use of paracetamol during pregnancy and the mechanistic uncertainties; however, to the best of our knowledge no prior study has performed a systematic disproportionality analysis restricted to paracetamol monotherapy in relation to autism spectrum disorder.

In the present study, we analyzed all FAERS data from January 2010 through September 2025 to characterize ASD-related reports associated with paracetamol, describe reporting patterns, and evaluate potential disproportionality signals using established pharmacovigilance algorithms.

## 2. Materials and Methods

### 2.1. Data Source

All publicly available FAERS (Silver Spring, MD, USA) records between January 2010 and 10 October 2025 were retrieved. FAERS collects spontaneous adverse event (AE) reports submitted by healthcare professionals, consumers, and manufacturers worldwide. Duplicate and follow-up entries were consolidated according to FDA guidance, retaining the most recent version for analysis.

### 2.2. Case Identification

Reports listing paracetamol (acetaminophen) as the primary or secondary suspect drug were extracted. Neurodevelopmental events were identified using Medical Dictionary for Regulatory Activities (MedDRA) (version 26.1, MedDRA Maintenance and Support Services Organization (MSSO), McLean, VA, USA) Preferred Terms (PTs) relevant to autism: Autism Spectrum Disorder, Autism, Childhood Autism, and Pervasive Developmental Disorder.

### 2.3. Data Classification

Variables captured included reporter type, patient sex and age, country, outcome, and seriousness. Reporter roles were grouped as consumer/non-professional versus healthcare professionals. Seriousness followed FDA definitions: death, life-threatening condition, hospitalization, disability, congenital anomaly, or other medically significant event.

### 2.4. Signal Detection

Descriptive statistics summarized case characteristics. Disproportionality analysis used Reporting Odds Ratio (ROR), Proportional Reporting Ratio (PRR), Information Component (IC), and Empirical Bayes Geometric Mean (EBGM), consistent with WHO–Uppsala Monitoring Centre (UMC) and FDA methodology. A signal was defined as: ROR 95% CI lower > 1; PRR ≥ 2 and χ^2^ ≥ 4; IC025 > 0; or EB05 > 2.

Given that 98.6% of ASD-related reports originated from consumers, reporter-type bias was anticipated. Therefore, analyses were exploratory and hypothesis-generating rather than confirmatory.

### 2.5. Ethical Approval

The FAERS database contains fully de-identified, publicly accessible data; therefore, this study did not require ethical approval or informed consent.

## 3. Results

In total, 172,129 FAERS reports involved paracetamol-containing products. After excluding cases listing other suspect or concomitant drugs, 1776 reports remained that included at least one ASD-related Preferred Term (PT) and paracetamol as the only reported drug.

Of these, 1751 (98.6%) were submitted by consumers and only 25 (1.4%) by healthcare professionals. A chi-square test comparing reporter type distributions revealed that consumer-submitted reports were significantly overrepresented among paracetamol–ASD cases (98.6%) relative to all ASD-related reports in FAERS (71.9%) (χ^2^ ≈ 1200, *p* < 0.0001).

All cases were classified as serious according to FDA criteria. The MedDRA term “fetal exposure during pregnancy” appeared in 1487 (83.8%) reports. Outcome data indicated that 1760 (99.1%) were coded as “other outcomes”, 6 (0.3%) as “disabled”, and 13 (0.7%) as “congenital anomaly”. No deaths were reported.

Age information was available for 21 cases ranging from 3 months to 36 years, with a median of approximately 5 years and a mean of 7.4 years (Table 1). Most cases corresponded to early childhood and school-age diagnoses, while only a few involved adolescent or adult patients.

**Figure 1:**
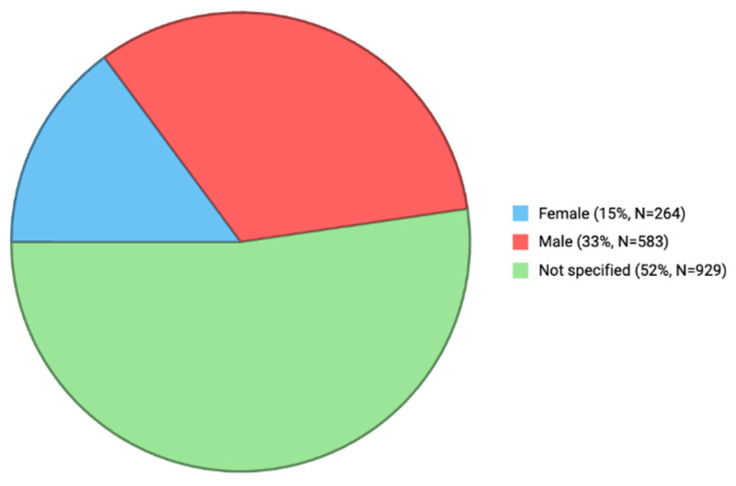
Gender distribution of ASD-related reports associated with paracetamol (FAERS, 2010–2025).

The gender distribution of ASD-related reports associated with paracetamol is shown in Figure 1. Gender information was available in 847 reports (47.7%); of these, 583 involved males and 264 females, while gender specification was lacking in 929 reports.

**Figure 2:**
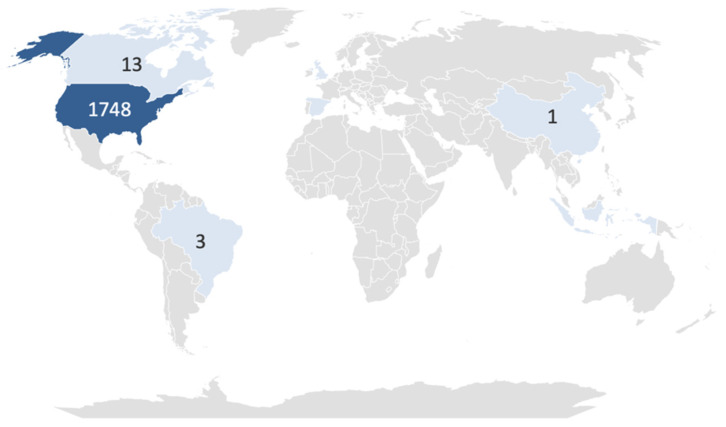
Geographic distribution of ASD-related reports associated with paracetamol (FAERS, 2010–2025).

The geographic distribution of ASD-related reports associated with paracetamol is presented in Figure 2. Most reports originated from the United States (n = 1748; 98.4%), followed by Canada (n = 13), the United Kingdom (n = 5), Brazil (n = 3), Spain (n = 2), China (n = 1), and Indonesia (n = 1). Three reports did not specify the country of origin.

**Figure 3:**
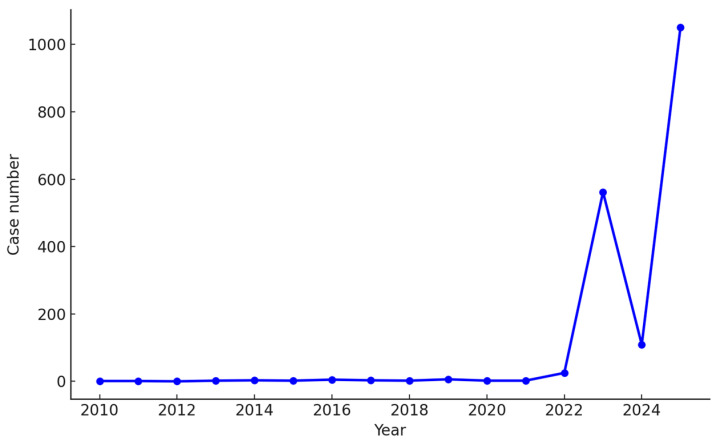
Yearly trend of autism spectrum disorder (ASD) reports associated with paracetamol in the FAERS database (2010–2025).

The yearly trend of ASD reports associated with paracetamol in the FAERS database between 2010 and 2025 is illustrated in Figure 3, demonstrating a sharp increase after 2022, with 1051 reports in 2025, 562 in 2023, and 109 in 2024, while earlier years each contributed fewer than 30 reports.

Disproportionality analysis for paracetamol-associated ASD reports in the FAERS is shown in Table 2.

## 4. Discussion

This pharmacovigilance analysis of FAERS between 2010 and 2025 identified 1776 reports linking paracetamol exposure to ASD terms after exclusion of concomitant drugs. All cases were classified as serious, and 83.8% explicitly included fetal exposure during pregnancy, supporting a predominantly prenatal exposure context.

Across all four disproportionality algorithms [ROR: 69.8, (95% CI 66.1–73.8), PRR: 69.2, IC025: 5.60, and EBGM = 50.4, (EB05–EB95: 48.3–52.6)] a strong and consistent positive signal was observed. While the convergence of frequentist and Bayesian estimators reinforces the internal consistency of the finding, these metrics reflect reporting disproportionality rather than causal risk.

### 4.1. Temporal Pattern and Reporter-Type Relationship

The temporal distribution of reports shows a sharp increase after 2022, which may coincide with heightened public discussion and media attention regarding prenatal paracetamol safety. Report counts rose dramatically from fewer than 30 reports per year before 2022 to 562 in 2023 and 1051 in 2025. Notably, 98.6% of ASD-related reports were submitted by consumers, while only 1.4% originated from healthcare professionals. The predominance of consumer-submitted reports appears particularly marked for paracetamol-related ASD cases, suggesting that this trend may reflect topic-specific public awareness or reporting bias rather than a genuine increase in clinically confirmed events. Such patterns are consistent with previous pharmacovigilance observations, where intense media attention or social discourse around a potential drug–outcome relationship amplified spontaneous consumer reporting. Consequently, this imbalance underscores the importance of cautious interpretation of spontaneous report data, especially for widely discussed and self-reported conditions such as ASD. This parallel rise in total report numbers and consumer-submitted cases might indicate that the post-2022 surge reflects increased information-driven public concern or social media-driven reporting due to information disorder rather than a true epidemiologic shift [31]. Similar patterns have been observed in FAERS following widely publicized safety debates, illustrating how notoriety bias may influence spontaneous reporting trends.

The exponential increase in reports observed in 2025 is unlikely to be explained by biological mechanisms or by incremental improvements in pharmacovigilance infrastructure. Instead, it closely aligns with specific sociopolitical events. As described by a recent work, political rhetoric in September 2025 urging women to avoid paracetamol framed its use as a matter of maternal morality, a narrative that likely precipitated a surge of retrospective consumer reporting [32]. This interpretation is consistent with the American College of Medical Toxicology’s assessment that a “storm of media attention,” compounded by litigation-driven narratives, distorted public perception and generated a substantial disconnect between the drug’s established safety profile and the volume of reported adverse events [33].

While FAERS remains a valuable tool for signal detection in pharmacovigilance, a clear understanding of its inherent limitations is essential for appropriate contextualization of findings [34]. The dramatic increase in reports observed in 2025 exemplifies a pattern of stimulated reporting rather than a true biological signal. As highlighted in recent analyses, this surge coincided with intense political discourse in the United States that framed paracetamol use as a moral failing [32], reinforcing the conclusion that external sociopolitical forces played a central role. The striking imbalance between consumer (98.6%) and healthcare professional (1.4%) reports further supports this interpretation. Healthcare professionals generally base clinical practice on scientific consensus, which does not support a robust causal association between paracetamol exposure and ASD [18,35], whereas consumer reporting is more susceptible to media narratives and litigation-related messaging. The American College of Medical Toxicology similarly concluded that the recent spike in reports reflects media- and litigation-driven influences rather than clinical observation. Taken together, the paucity of healthcare professional reports may be viewed as a functional negative control, reinforcing the conclusion that the observed reporting surge is primarily social in origin rather than clinical [33].

### 4.2. Demographic and Epidemiologic Context

Age information was available for 21 reports, ranging from 3 months to 36 years (median ≈ 5 years, mean ≈ 7.4 years). The majority of ASD diagnoses occurred between 2 and 8 years of age, consistent with the CDC’s ADDM Network median diagnostic age of 47 months (≈3.9 years) [3] and WHO’s estimated 3–5-year diagnostic window [2]. A small number of reports involved adolescents or adults, which may reflect delayed diagnosis or retrospective parental reporting. Given the predominance of pregnancy-related terms, this temporal pattern aligns with prenatal exposure followed by later childhood diagnosis.

Gender information was available for 847 reports (47.7%), with 583 (68.8%) male and 264 (31.2%) female cases (male:female ≈ 2.2:1). Although this ratio is lower than the ≈3–3.4:1 range reported in large-scale population studies [2,3], the trend still indicates male predominance consistent with established ASD epidemiology. The missing gender data (52.3%) likely reflect underreporting rather than true deviation from known patterns.

### 4.3. Geographic Distribution and Environmental Context

Nearly all reports originated in the United States (98.4%), with smaller numbers from Canada, the United Kingdom, Brazil, Spain, China, and Indonesia. This pattern likely reflects differences in pharmacovigilance awareness and data submission activity but may also align with broader environmental and industrial factors.

While paracetamol use during pregnancy has received considerable attention, ASD is a multifactorial condition influenced by genetic and environmental determinants. Regional variations in ASD reporting may therefore reflect differences in overall environmental chemical burden rather than medication exposure alone. Countries contributing the majority of FAERS reports (primarily industrialized regions) are also those with the highest measured emissions of neurotoxic and endocrine-active pollutants, including heavy metals, phthalates, BPA, and pesticides [29,36]. Thus, the geographic clustering observed here could partly correspond to industrial density, environmental pollution, or differential monitoring capacity, rather than differential pharmaceutical risk.

This perspective emphasizes that pharmacovigilance signals should be interpreted within their broader environmental and toxicological context. Integrating spontaneous report data with biomonitoring and geochemical exposure surveillance systems such as National Health and Nutrition Examination Survey (NHANES), Human Biomonitoring for Europe (HBM4EU) and United Nations Environment Programme (UNEP) global datasets could clarify whether ASD reporting intensity parallels environmental contaminant exposure or not. Such integration would help determine whether apparent regional differences stem from medication use, environmental pollution, or their potential combined effects.

### 4.4. Methodological Considerations and Congenital Anomaly Data

Spontaneous reporting databases such as FAERS inherently lack denominator data, validated diagnoses, and exposure quantification, which precludes causal inference. The predominance of consumer-submitted cases and the surge in reporting after 2022 suggest awareness-driven inflation rather than true incidence change.

Thirteen reports (0.7%) included the MedDRA term congenital anomaly, a rate well within expected background variability. Large cohort studies found no association between maternal paracetamol exposure and overall or specific congenital malformations [37]. Therefore, these FAERS entries likely represent background reporting rather than evidence of a teratogenic effect.

We acknowledge the inherent limitations in data reliability associated with consumer-generated reports. As described by Potter et al. (2025), consumer submissions to FAERS often lack medical verification and are susceptible to recall bias, incomplete clinical context, and outcome misclassification [34]. In the context of the present analysis, however, this limitation also constitutes a central finding: the observed disproportionality signal is driven almost exclusively by unverified consumer reports that appear to be stimulated by public notoriety, whereas the relative absence of reporting by healthcare professionals is concordant with the lack of causal evidence in high-quality epidemiological studies [35].

A further critical limitation of FAERS is the absence of information on key confounders, including maternal health status, underlying indications for paracetamol use, environmental exposures, and genetic susceptibility, all of which preclude causal inference. The implications of these missing variables have, however, been clarified through alternative epidemiological approaches. In particular, an umbrella review evaluated sibling-controlled cohort studies, which inherently adjust for shared genetic and familial environmental factors. The finding that the apparent association between prenatal paracetamol exposure and ASD disappears in these methodologically robust designs strongly suggests that the disproportionality signal observed in FAERS primarily reflects residual confounding—such as confounding by indication or genetic pleiotropy—rather than a direct pharmacological effect [35].

### 4.5. Interpretation and Biological Plausibility

The disproportionality metrics presented in Table 2 reveal an exceptionally strong signal that remains consistent across frequentist and Bayesian algorithms. Although the magnitude of disproportionality greatly exceeds relative risks reported in cohort or case–control studies (typically 1.2–1.6), the observed patterns—stronger signal for prenatal exposure and male predominance—highlight consistent reporting trends rather than establishing causality. Experimental models demonstrate rapid placental transfer, fetal brain penetration, and mechanistic pathways including oxidative stress, glutathione depletion, mitochondrial dysfunction [25], neuroinflammatory activation, and epigenetic modification of neurodevelopmental genes [6,12,13,38]. Nevertheless, a structured literature review of animal models found that most studies did not account for the original indication of use (e.g., fever or pain) and revealed wide heterogeneity in dose and duration of exposure, pointing to relevant gaps in translation of preclinical findings to human neurodevelopmental risk [39]. Together, these provide a coherent framework for hypothesis generation but remain insufficient to establish causality in humans [40].

Furthermore, the discrepancy between the strong disproportionality signal observed in FAERS and the epidemiological evidence base is underscored by a recent umbrella review [35]. While conventional cohort studies have reported associations between prenatal paracetamol exposure and neurodevelopmental outcomes, high-quality sibling-controlled analyses—which inherently account for shared familial, genetic, and environmental confounders—consistently demonstrate attenuation of the reported risks for ASD and ADHD toward the null. This pronounced contrast supports the interpretation that the disproportionality signal detected in FAERS is more likely an artifact of stimulated reporting than an indicator of true biological risk.

The divergence between the magnitude of the statistical signal (ROR = 69.8) and the absence of corroborating causal evidence illustrates a well-recognized limitation of spontaneous reporting systems. As discussed in our previous pharmacovigilance analyses on breastfeeding safety [41,42], FAERS functions as a highly sensitive screening tool for signal detection; however, adverse events reported in this context are not synonymous with adverse drug reactions. This distinction is further emphasized by Potter et al. (2025), who highlight that disproportionality signals require careful contextualization before any causal interpretation [34]. Within this framework, the umbrella review by Sheikh et al. (2025) reinforces the conclusion that the observed signal is primarily driven by reporting dynamics, including notoriety bias, rather than biological causality [35].

Although experimental models have proposed potential mechanisms—such as oxidative stress or disruption of the endocannabinoid system—the translational relevance of these findings to human pregnancy remains uncertain. A recent Quantitative Weight-of-Evidence (QWoE) assessment, which systematically evaluated 188 endpoints across 34 in vivo studies, concluded that there is no consistent evidence of developmental neurotoxicity at therapeutic or non-systemically toxic doses [28]. Reported adverse outcomes were predominantly observed at suprapharmacological exposure levels associated with systemic toxicity, limiting their relevance to human therapeutic use.

Moreover, within the PATH translational framework, robust inference requires a coherent evidentiary chain linking preclinical mechanisms to human pathophysiology [43]. At present, this chain remains incomplete for paracetamol: mechanistic signals identified in rodent models, including oxidative stress, have not been validated as causal drivers of neurodevelopmental disorders in humans at clinically relevant doses. Consequently, the biological plausibility of a causal association remains weak. To address this translational gap, future research should move beyond direct extrapolation from animal models. The integration of systems biology approaches and the development of humanized computational models informed by preclinical data, as advocated in recent translational guidelines [43], may provide a more rigorous framework for evaluating potential risks. Nevertheless, based on the current weight of evidence, available preclinical data do not support the hypothesis that therapeutic paracetamol use causes ASD or ADHD [28].

### 4.6. Clinical Implications

From a clinical standpoint, these findings do not justify changes in current therapeutic recommendations but highlight the need for cautious prescribing. Both ACOG and the European Medicines Agency (EMA) continue to recommend paracetamol as the preferred antipyretic and analgesic during pregnancy when clinically indicated [18,44]. Nonetheless, evidence of maternal–fetal transfer [22] and potential neurodevelopmental vulnerability [15,25] underscore the importance of using the lowest effective dose for the shortest duration [18]. Healthcare providers should counsel patients that existing epidemiologic associations between prenatal paracetamol exposure and neurodevelopmental outcomes do not establish causality but warrant ongoing pharmacovigilance and translational research. More robust studies, like cohort-based or registry-based research, and a careful exploration of confounders, would be necessary to draw firmer conclusions regarding the relationship between prenatal paracetamol exposure and autism.

### 4.7. Strengths and Limitations

To the best of our knowledge, this study provides the first large-scale pharmacovigilance analysis focusing on paracetamol monotherapy and ASD-related reports during pregnancy, using over fifteen years of FAERS data and multiple disproportionality algorithms (e.g., ROR, PRR, IC, EBGM) to ensure robustness. The study was conducted in accordance with the “REporting of A Disproportionality analysis for drUg Safety signal detection using individual case safety reports in PharmacoVigilance (READUS-PV)” guidelines, which outline methodological and reporting standards for pharmacovigilance database analyses [45]. However, the disproportionality analysis could not be stratified by reporting region, as nearly all cases originated from the United States. Additional limitations include underreporting, reporting bias, and the absence of key variables such as dosage, duration of exposure, concomitant drugs, family history, environmental exposures, and genetic susceptibility factors which preclude adjustment for major confounders. Finally, while this study identifies a temporal correlation between the reporting surge and public events, a granular mechanistic analysis of social media algorithms and information propagation patterns lies beyond the scope of pharmacovigilance. Although a recent work provides a framework for understanding the sociopolitical triggers (e.g., purity rhetoric) [32], we suggest that future interdisciplinary research applying communication science methodologies is needed to fully map the ‘root causes’ of such digital disinformation waves in adverse event reporting.

## 5. Conclusions

In summary, our FAERS-based analysis identified a consistent disproportionality signal between paracetamol exposure and ASD-related reports, predominantly during pregnancy. However, this signal must be interpreted strictly as hypothesis-generating rather than causal, given the well-recognized limitations of spontaneous reporting systems, including reporting bias and incomplete case information. Critical confounders were unavailable in FAERS, such as family history of autism, concomitant medication use, autism-associated genetic variants (e.g., SHANK, NRXN, CNTNAP2, NLGN), as well as dose, duration of exposure, and relevant environmental factors. In the absence of these data, no definitive conclusions regarding neurodevelopmental risk can be drawn. The marked discrepancy between the strong FAERS disproportionality signal and the null findings consistently reported in robust epidemiological studies—particularly sibling-controlled designs—suggests that the observed signal is more likely driven by stimulated reporting and public concern (notoriety bias) than by a true biological effect. Accordingly, FAERS should be regarded as a sensitive screening tool for signal detection, not as evidence of causality. Clarification of the neurodevelopmental safety profile of prenatal paracetamol exposure will require integrated approaches combining pharmacovigilance with well-designed birth-cohort epidemiology, biomarker-based investigations, and mechanistic research. In conclusion, regulatory decision-making should prioritize triangulation between advanced translational models (humanized computational systems) and high-quality epidemiological data, rather than relying on isolated spontaneous reporting signals prone to external biases.

## Figures and Tables

**Table 1:** Age distribution of ASD-related reports associated with paracetamol (FAERS 2010–2025).

Age Range (Years)	Number of Cases	Percentage (%)
<2 years	5	23.8
2–5 years	7	33.3
6–12 years	4	19.0
≥13 years	5	23.8
Total	21	100

**Table 2:** Disproportionality metrics for paracetamol-associated ASD reports in the FAERS database (2010–2025).

Disproportionality Metrics	Value	95% Confidence Interval (CI)	Number of Cases	Signal Status
Reporting Odds Ratio (ROR)	69.8	66.1–73.8	5	Positive signal
Proportional Reporting Ratio (PRR)	69.2	—	7	Positive signal
Information Component (IC)	5.65	IC025 = 5.60	4	Positive signal
Empirical Bayes Geometric Mean (EBGM)	50.4	EB05 = 48.3, EB95 = 52.6	5	Positive signal

## Data Availability

The raw data supporting the conclusions of this article are available from the authors upon reasonable request.
